# Challenging Hazards Amidst Observational Simulation in the Emergency Department: Advancing Gamification in Simulation Education Through a Novel Resident-led Skills Competition

**DOI:** 10.7759/cureus.3563

**Published:** 2018-11-08

**Authors:** Nicholas Salerno, Dimitrios Papanagnou, Priyha Mahesh, Kaitlin M Bowers, Scott H Pasichow, Sara Paradise, Xiao Chi Zhang

**Affiliations:** 1 Emergency Medicine, Louisiana State University Health Sciences Center, New Orleans, USA; 2 Emergency Medicine, Thomas Jefferson University, Philadelphia, USA; 3 Miscellaneous, Thomas Jefferson University, Philadelphia, USA; 4 Emergency Medicine, Doctors Hospital North, Columbus, USA; 5 Emergency Medicine, Alpert Medical School of Brown University, Providence, USA; 6 Emergency Medicine, University of California, Irvine, USA

**Keywords:** emergency medicine, simulation competition, innovation, gamification, medical education, medical simulation

## Abstract

Medical simulation competitions have become an increasingly popular method to provide a hands-on “gamified” approach to education and training in the health professions. The most well-known competition, SimWars, consists of well-coordinated teams that are tasked with completing a series of mind-bending clinical scenarios in front of a live audience through ‘bracket-style’ elimination rounds. Similarly, challenging hazards amidst observational simulation (CHAOS) in the emergency department (ED) is another novel approach to gamification in both its structure and feel. Conducted at the Council of Emergency Medicine Resident Directors (CORD) 2018 National Assembly in San Antonio, Texas, instead of assigning premeditated teams, it placed random Emergency Medicine (EM) faculty, residents, and medical students together in teams to test them on a variety of fundamental EM content areas. Additionally, the event incorporated multiple levels within each round, allowing the inclusion of additional information to be shared with participants to support “switching gears,” as is typical for teams working in the ED and augmenting the perceived level of “chaos.”

To assess this pilot project, formal quantitative and qualitative feedback was solicited at the end of the session. Quantitative evaluation of the intervention was obtained through an eight-item questionnaire using a five-point Likert-type scale from 19 of the 20 enrolled participants (95% response rate). Responses were generally positive with an overall course rating score of 4.45 out of 5 (SD +/- 0.62). Qualitative feedback revealed that learners enjoyed performing procedures and networking with their EM colleagues. The majority of residents (95%) recommend the activity be integrated into subsequent conferences. Areas for improvement included shorter cases and minimizing technical malfunctions.

CHAOS in the ED was a successful pilot study that incorporated gamification as a means to deploy simulation-based training at a national emergency medicine conference in a community of simulation educators. Future studies should focus on incorporating learners’ feedback into subsequent CHAOS iterations and reducing overhead costs to increase its adoption by both regional and national audiences.

## Introduction

The rise in team-based simulation as a training modality reflects healthcare’s shift in adopting practice-based educational methods [1]. The opportunity for practice-based education is of high importance in the emergency department (ED), as stress and clinical ambiguity challenge clinical teams and patient safety. Simulation-based training is routinely implemented in emergency medicine (EM) post-graduate residency training programs, as it provides residents with a psychologically safe learning environment for them to make mistakes and hone their skills in a setting that replicates the clinical environment. Simulation allows for reflection and the development of critical thinking, psychomotor, and interpersonal skills; it also provides an opportunity for the objective evaluation of trainees’ skillset [1-5]. Procedural, clinical, and teamwork skills developed during simulation training have been shown to directly transfer into clinical practice and potentially reduce medical errors while improving increasing clinical performance [6,7].

While simulation training has become a cornerstone in training across EM residency programs in the country, simulation-based competitions, such as SimWars®, have successfully brought this innovative learning method to the national stage. In SimWars®, teams undertake a simulated medical scenario, receives a public debriefing by the judges, and can advance to the next level based on audience polling. The combination of input from audience participation and an expert panel allows for a fun, interactive approach that tests residents’ core competencies [8].

The concept of public performance and evaluation during the debriefing may imply a psychologically unsafe educational design; research, however, suggests that knowledge is better retained during stressful situations [9]. Surveys distributed after these competitions show that both mentors and trainees agree on the benefits of these simulations [10]. Responses from residents suggest that participation helped them identify strengths and weaknesses, and contributed toward their education [8,11].

The purpose of this study was to implement and assess a new skills competition organized by the Emergency Medicine Residents’ Association (EMRA): challenging hazards amidst observational simulation (CHAOS) in the ED. In contrast to previous simulation-based competitions, most notably SimWARS®, where teams of residents are gathered from various EM residency program to compete in teams through a series of simulated medical cases, the authors elected to design a more authentic, chaotic, multi-tiered approach to a simulation-based skills competition. By incorporating conceptual frameworks from popular television shows (i.e., team-building components of “Escape Rooms”, resource utilization from “Guy’s Grocery Games”, cognitive decision-making processes from “Family Feud” and navigating through insurmountable obstacles from “Legends of the Hidden Temple”), the investigators developed a multi-tiered skills competition for teams of randomly assigned EM physicians that competed in a series of fast-paced, interactive gaming activities that tested their skills in EM skills during the Council of Emergency Medicine Resident Director (CORD) 2018 National Assembly, which was hosted in San Antonio, Texas. Each skill challenge was created through a joint-collaborative effort among the seven interdisciplinary EMRA committees (i.e., wilderness, sports medicine, prehospital, EMS, toxicology, critical care, and ultrasound).

## Technical report

Study setting

Learners Targeted

Medical students, EM residents, and EM faculty members.

Group Size

Four participants per team, up to eight teams (32 participants total).

Personnel

Each team required four volunteers (i.e., medical students or residents) and one judge (i.e., resident, fellow, or faculty) for a total of 20 volunteers and five judges. The volunteers were responsible for setting up the stage for each round and directing participant traffic. The judge was responsible for scoring each team’s performance based on several pre-identified variables, including clinical accuracy, speed, and the number (percent) of critical actions completed. Judges were provided with a scoring card and accompanying rubric for each round. The entire session was facilitated by two individuals to both announce the events and engage the audience throughout all rounds.

Materials

The following audiovisual (AV) materials were required: two projectors, two screens, five handheld microphones, two stands (computer/projectors), speakers, and a timer. For the session activities, the following materials were required: bulky clothes, paracord (or a rope), trauma shears, duct tape, wood (for splinting), standard splinting/casting materials (i.e., plaster, cast padding, ACE bandages, a map of the region showing nearby hospitals, triage tags, paper clips, an intubation kit, endotracheal tubes, elastic boogies, and laryngoscope blades). Several low-fidelity simulation models were required; these include a TruCorp AirSim Standard Adult and Simulaid Pediatric airway task trainers. Required high-fidelity simulation trainers included: SonoSim LiveScan, SimuLab PacerMan, and FemoraLineMan systems.

Funding

CHAOS in the ED was sponsored by the Emergency Medicine Resident Association (EMRA) and the American College of Emergency Physicians (ACEP) Physician’s Evaluation and Educational Review (PEER), with equipment provided in-kind by the American College of Osteopathic Emergency Physicians (ACOEP), SimuLab, SonoSim, and FUJIFILM Sonosite during the competition [12-14].

Accreditation Council for Graduate Medical Education (ACGME) Milestones

The activity provided learners with the opportunity to deliberately practice several EM milestones, including patient care (i.e., multitasking, airway management, emergency stabilization, goal-directed focused ultrasound, vascular access), medical knowledge and interpersonal communication skills (i.e., team management) (Table 1).

**Table 1:** Applicable emergency medicine milestones for CHAOS in the ED activities per Accreditation Council for Graduate Medical Education (ACGME). CHAOS: Challenging hazards amidst observational simulation; ED: Emergency department; PC: Patient care; MK: Medical knowledge; ICS: Interpersonal communication skills. A milestone is a medical specialty-specific element that guides the assessment of the resident-physicians through the six core competencies in residency training (patient care, medical knowledge, practice-based learning and improvement, interpersonal and communication skills, professionalism, and systems-based practice). Each core competency may contain a different number of elements, identified with a numeric number. For example, PC8 is the eighth element in the emergency medicine (EM) ACGME patient care milestone that evaluates the resident on his ability to multitask. Each medical specialty has different numbers of elements in the core competency. This table describes relevant EM ACGME milestones for each CHAOS in the ED activity.

CHAOS Round	Activities	Relevant Emergency Medicine ACGME Milestones
Pre-Event Selection	Clinical Question Card	MK – Medical Knowledge
Round 1A	Wilderness Emergencies: stabilization of orthopedic injuries	PC1 – Emergency Stabilization; PC3 – Diagnostic Studies; PC13 – Wound Management; ICS2 – Team Management
Round 1B	Tabletop Mass Casualty Drill: toxicological exposures and traumatic injuries	PC1 – Emergency Stabilization; PC7 – Disposition; ICS2 – Team Management
Round 2A	Critical Care Management: central venous line access, arterial line access, transvenous pacing	PC1 – Emergency Stabilization; PC4 – Diagnosis; PC6 – Observation and Reassessment; PC8 – Multi-tasking; PC9 – General Approach to Procedures; PC12 – Goal-directed Focused Ultrasound; PC14 – Vascular Access; ICS2 – Team Management
Round 2B	Rapid-Fire Ultrasound Diagnosis	PC3 – Diagnostic Studies; PC12 – Goal-directed Focused Ultrasound; ICS2 – Team Management
Round 3	Airway Challenge: securing challenging airway in space-confining situation	PC8 – Multi-tasking; PC9 – General Approach to Procedures; PC10 – Airway Management; ICS2 – Team Management

Detailed activity description

A four-hour pilot study of an EM-themed medical simulation contest based on numerous popular gamification frameworks was developed and implemented during the Council of Emergency Medicine Resident Director (CORD) 2018 National Assembly hosted in San Antonio, Texas. Specific competition challenges were selected from a joint collaboration with the EMRA committee chairs, including, but not limited to, critical care medicine, toxicology, pre-hospital and disaster medicine, simulation, wilderness, ultrasound, and education. This event was advertised on the EMRA website [15] and various social media outlets (i.e., Facebook, Twitter) two months prior to the event.

Throughout the CORD conference in the days leading up to the competition event, organizers recruited eligible participants interested in competing by having them fill out a 'question card.' These cards contained a multiple choice clinical question (provided by ACEP PEER), basic demographics questions, and space to indicate one’s level of training (i.e., medical student, attending, resident). These cards must be filled out in the presence of, and collected by the event organizer upon completion. The cards were later graded and separated based on whether the clinical questions were correctly answered. All eligible participants who submitted a question card were instructed to show up at the event to see if they would be selected to compete. The final teams were selected via a lottery system, drawing cards at random from the “correct” pile just prior to the start of the event, until eight teams of four were assembled. If there were fewer than 32 eligible participants present on the day of competition, the teams were assembled at random by the event coordinators to ensure the maximum of four participants-teams.

**Figure 1:**
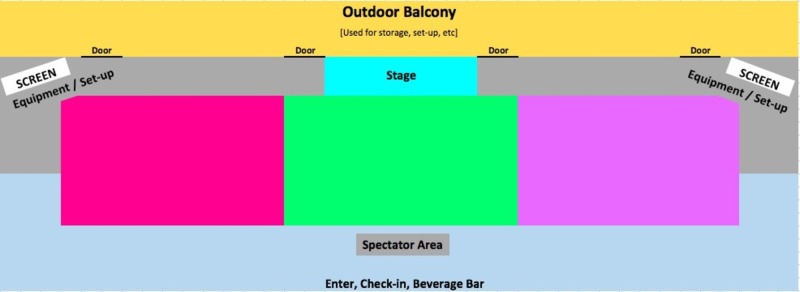
Schematic illustration of the CHAOS arena. CHAOS: Challenging hazards amidst observational simulation.

Once teams were determined, they were directed to the CHAOS arena, where they were expected to utilize teamwork, communication, task-delegation, critical thinking, attention to detail, as well as lateral thinking to tackle waves of increasingly complex tasks, ranging from patient stabilization, transport, splinting, intubation, central venous access, and transvenous cardiac pacing. Figure 1 illustrates a simplified diagram of the CHAOS in the ED event, with the three colored rectangular sections (magenta, green, pink) demonstrating where individual teams can participate simultaneously during each event. All participants were encouraged to observe and provide support to their peers throughout the event. Teams that were scheduled to complete identical same events were directed to a private adjacent room in order to prevent an unfair advantage.

**Figure 2:**
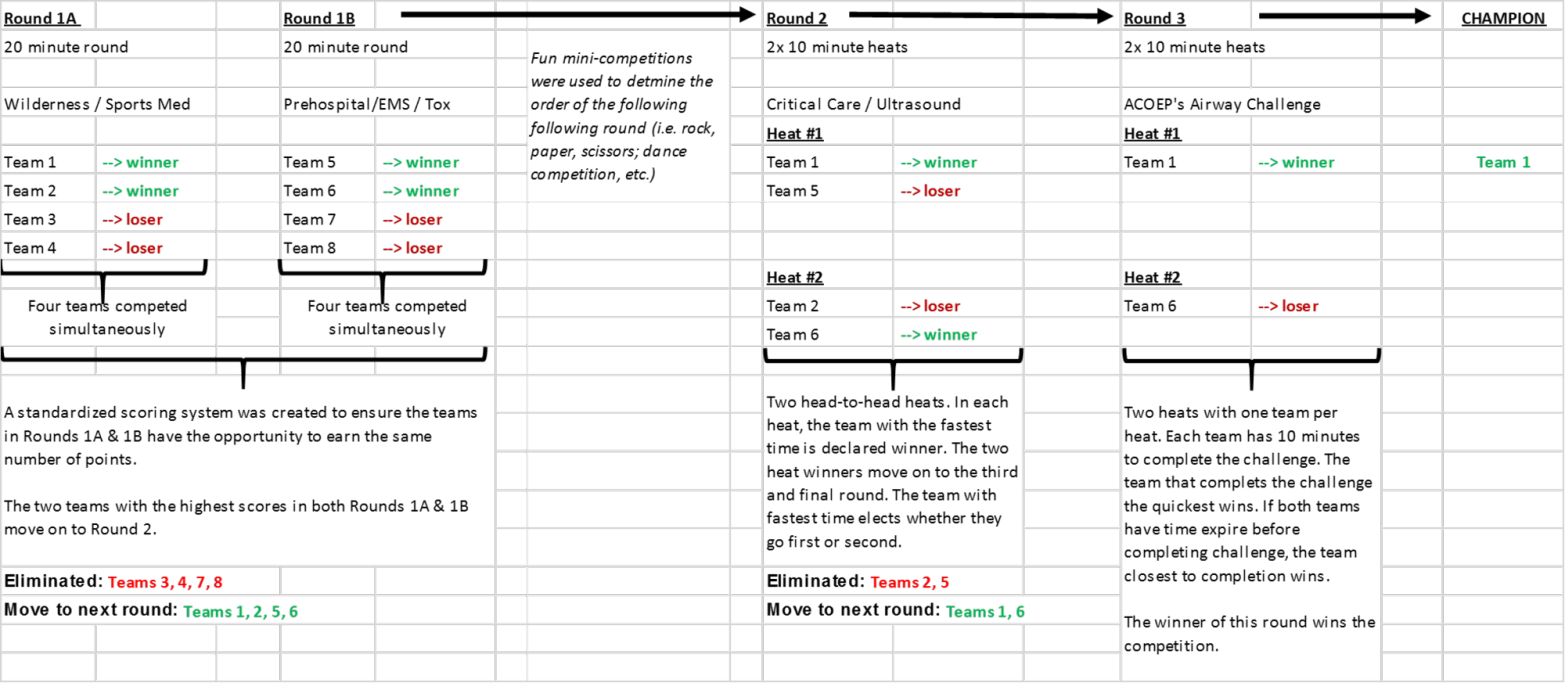
Potential workflow for a maximum of eight teams involved in CHAOS in the ED. Half of the teams participated in Round 1A, while the other half participated in Round 1B. The winning teams from Rounds 1A and 1B advanced to Round 2. The winners of Round 2 will proceed to Round 3 where one team is declared the winner. CHAOS: Challenging hazards amidst observational simulation; ED: Emergency department.

CHAOS in the ED was broken into three separate rounds (Rounds 1A, 1B, Round 2, and Round 3), each with EM-related tasks and challenges. Figure 2 demonstrates a potential workflow for a maximum of eight teams. At random, half of the teams were selected to participate in Round 1A. Following this round the remaining teams participated in Round 1B. The winning teams from Rounds 1A and 1B advanced to Round 2.

**Figure 3:**
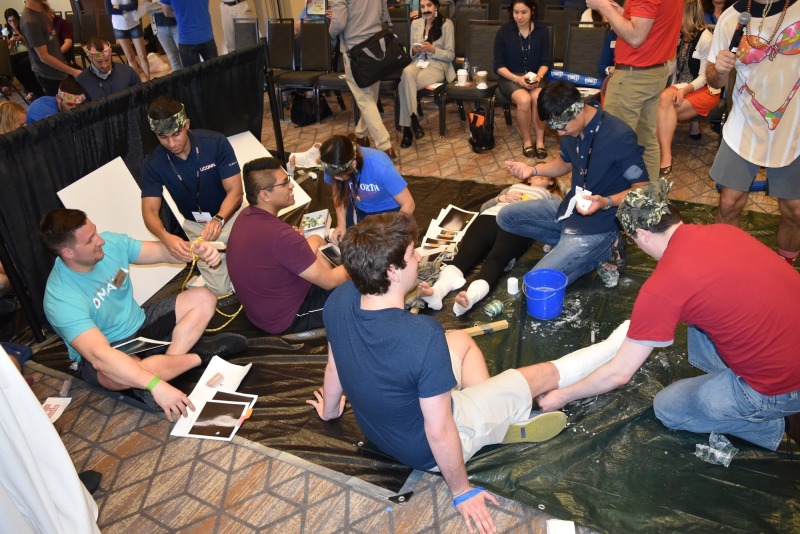
Participants are seen splinting a broken leg while matching X-ray images to injured patients as part of Round 1A.

Round 1A involved responding to injured hikers/volunteers after a bridge malfunction, and transporting them to the nearest hospital. Triage cards were placed in front of the injured hiker/volunteers that asked participants to describe any suspected injuries, as well as list the appropriate radiographs they would require for their suspected injuries. Round 1A continued in the hospital, where participants were asked to match a series of fracture names with the corresponding, correct radiograph from a stack of plain radiographs (Figure 3). They were then expected to carefully splint the fractures. Injuries included calcaneal fractures, anterior shoulder dislocations, forearm, and lower leg fractures, as well as cervical spine and pelvic fractures. Applicable procedures included: bulky jones dressing, shoulder reduction, thumb spica splint, and posterior ankle splint. The overall score was determined based on a performance checklist assessing for 1) primary survey; 2) appropriate physical examination; 3) fracture stabilization; 4) fracture reduction (when appropriate); 5) fracture identification via plain film; 6) splinting; and 7) time to completion.

**Figure 4:**
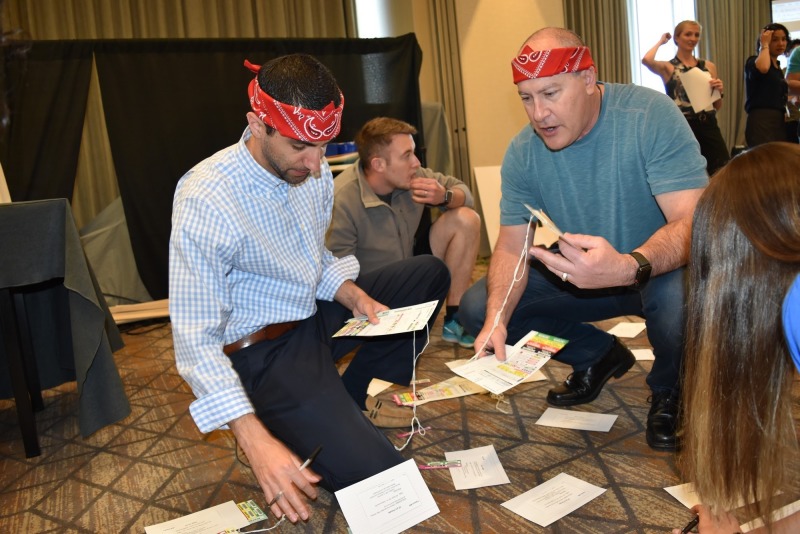
Participants working to triage simulated patients (with patient descriptions written on index cards) as part of Round 1B.

Round 1B involved each team rapidly triaging and treating victims of a mass casualty event (i.e., several oxygen tanks on a bus exploded outside of a secret government facility storing unknown volatile chemicals). This event was facilitated as a table-top game, where each patient was represented by cards with images of victims on the backs, laying on the ground at the start of the case. Each team was expected to implement the simple triage and rapid treatment (START) method to appropriately triage blunt trauma and toxicological injuries (Figure 4). The START method is used during mass casualty incidents where victims are quickly assessed and triaged with a colored tag that corresponds to the severity of their injury (i.e., deceased/expectant - black tag; immediate - red tag; delayed - yellow tag; and walking wounded/minor - green tag). Teams evaluated and triaged patients based on the severity of their injury, and wrote down the critical actions they would execute (i.e., medication administration, imaging tests ordered, antidotes-given, consults requested). For example, if the case is toxicologic in nature, the appropriate treatment/antidote must have been written on the card. Teams were then prompted to transport patients to the hospital with only a limited supply of ambulances. Ambulances showed up periodically, and points were awarded for the number of patients who arrived at the hospital in the time allotted. Teams were also scored on the quality of their hand-off report utilizing the SBAR (i.e., situation, background, assessment, and recommendation) format.

**Figure 5:**
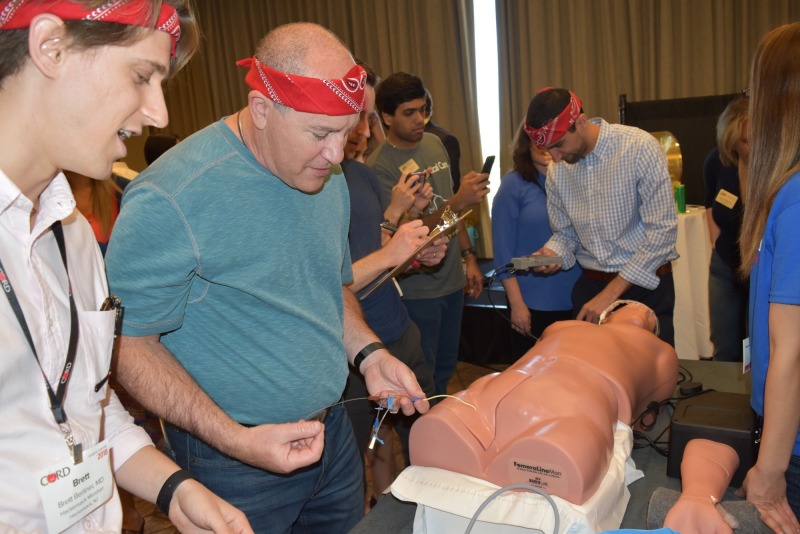
Participants are working to place a right femoral central line, right transvenous pacing as part of Round 2A.

**Figure 6:**
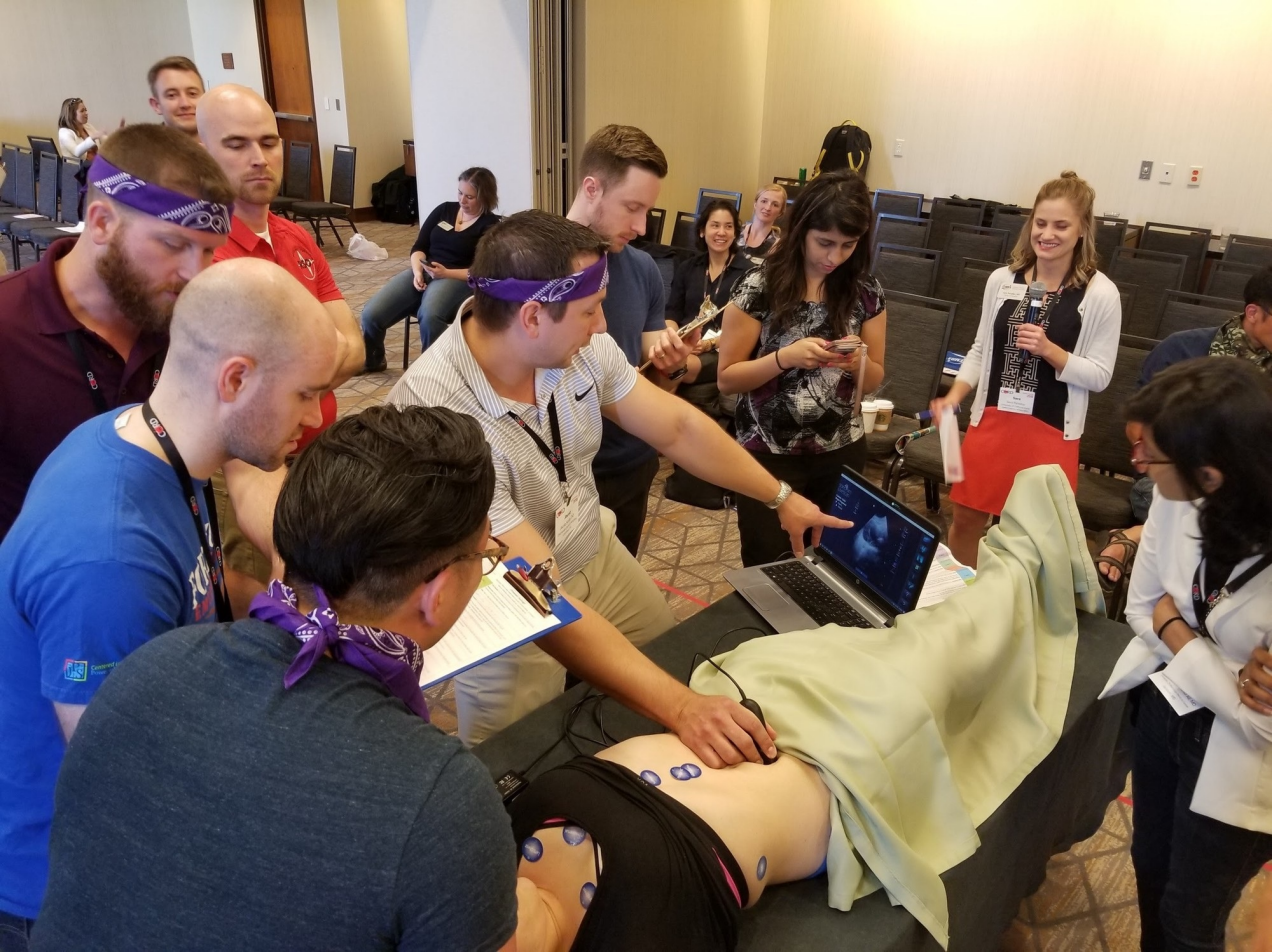
Participants using FUJIFILM SonoSim live scan technology to ultrasound pathologic images on a real patient used specially designed electronic stickers as part of Round 2B. Images of an aorta can be seen on the screen.

Round 2A challenged the four winning teams from Round 1 (two teams from Rounds 1A and two teams from 1B) to complete a set of critical care procedures (i.e., ultrasound-guided internal jugular transvenous pacer wire placement, femoral vein triple-lumen catheter placement, and radial artery line placement) in a simulated, critically-ill patient using low-fidelity task trainers within five minutes (Figure 5). Round 2B challenged the same group of learners to rapidly and correctly visualize, diagnose, and identify ultrasound images on an ultrasound task trainer (Figure 6). Each team was given five minutes to review up to six case vignettes with accompanying ultrasound images (i.e., pneumothorax, ectopic pregnancy, hypertrophic cardiomyopathy, abdominal aortic aneurysm, hemoperitoneum). Bonus points were offered to the team that finished first.

**Figure 7:**
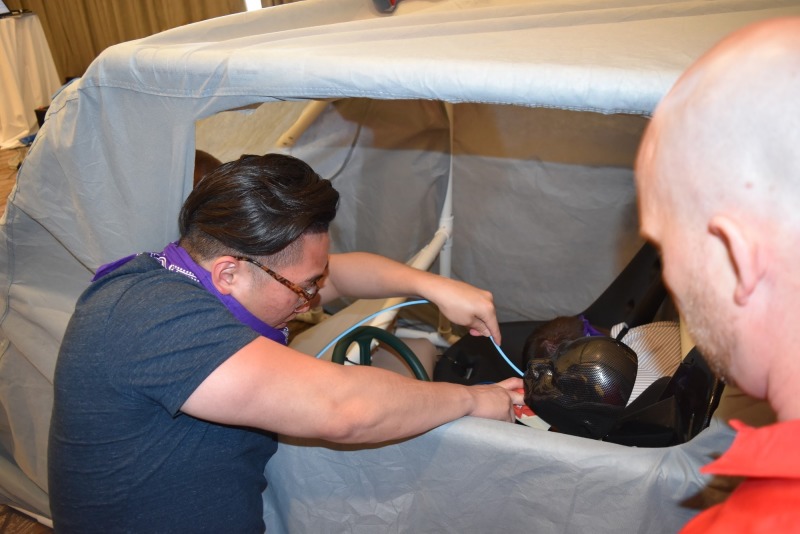
Round 3 involved intubation of simulated patients in a vehicle. Shown here is the PVC pipe made car, a simulated patient being intubated with a boogie and digital intubation technique.

Finally, Round 3 challenged the two teams with the highest scores from Round 2 to secure definitive airways in four simulated patients trapped in a motor vehicle accident with limited equipment. Each team member was tasked with intubating one of the four challenging airway task trainer models without using the same laryngoscope blade more than once. A standard sized car was designed and constructed from an assembly of PVC piping. Two race car bucket seats were purchased for use as the front seats, while the back bench seat was reproduced by using chairs from the conference room. Seats were tied to the PVC framework, which was then covered with a vinyl car cover. The car cover was modified to have two open windows (i.e., driver window and rear passenger side), and two small flaps in the windshields for judges to look into the car and assess intubation skills. A steering wheel was also added to further confine the space available. In order to successfully access the simulated patients, team members were to climb through the open windows to intubate in atypical positions (Figure 7). Available equipment included: four bag valve masks (BVMs), four endotracheal (ET) tubes of different sizes, one elastic boogie, three laryngoscope blades, and one laryngoscope handle. Since only one laryngoscope handle was available, team members were prompted to practice successful communication skills to properly identify which airway adjuncts were appropriate for each patient before passing the laryngoscope handle to other members. Round 3 concluded once all four patients were successfully intubated. The scenario was reset for each team. The team with the fastest time to completion was announced as the winner. The winning group received a one-year subscription to an online question bank and a customized “CHAOS in the ED” wireless phone charging station.

All participants were asked to complete the post-study survey evaluation. This study was exempted through the Thomas Jefferson Institutional Review Board.

Results

**Figure 8:**
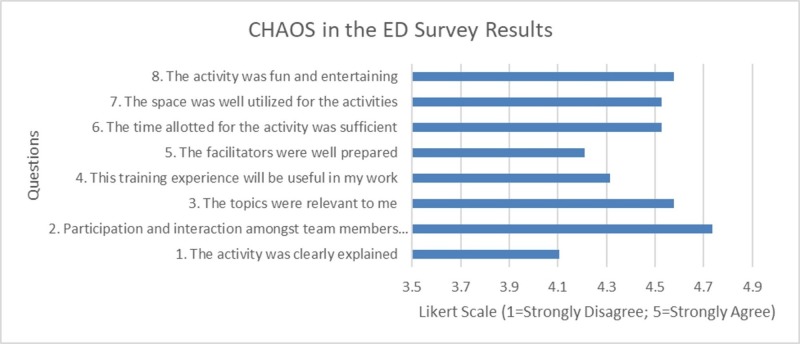
Survey response from CHAOS in the ED. CHAOS: Challenging hazards amidst observational simulation; ED: Emergency department.

Quantitative evaluation of the educational intervention was obtained through an eight-item questionnaire (derived from the home-institution post-course evaluation form) using a five-point Likert scale (1 = strongly disagree to 5 = strongly agree) from 19 of the 20 enrolled residents (95% response rate). The majority of respondents were post-graduate year (PGY)-2 (n = 5) and PGY-3 residents (n = 8), with additional representation from two PGY-1 residents, one medical student, one fellow, and one attending (Table 2). Responses were positive, with an overall activity rating score of 4.45 out of 5 (SD +/- 0.62) (Figure 8). Nine out of 19 participants have either participated or observed a simulation competition in the past, and only two participants reported having previous clinical experiences before the CHAOS competition. Nearly all learners appreciated the efficiency of the CHAOS competition with relevant and entertaining EM-challenges that encouraged team member interactions with respective average Likert scores over 4.5. Qualitative feedback identified learners’ appreciation of the clinical relevance of the simulation, the opportunity to networking, being able to participate in the dynamic and chaotic atmosphere, and being able to work with residents from different programs. Specific areas for improvement included requests for additional explanations of each activity, additional facilitator preparation, earlier sign-up, and the inclusion of additional mass-casualty events. Overall, 53% (n = 10) of respondents reported improved levels enthusiasm after completing the event, and an overwhelming majority of participants (95%) would like this competition to be repeated in future EM national conferences.

**Table 2:** Level of training for CHAOS in the ED participants. CHAOS: Challenging hazards amidst observational simulation; ED: Emergency department; MS: Medical student; PGY: Post graduate year (resident physician).

Respondent Types	Total #	Percentage
MS	1	5.26%
PGY 1	2	10.53%
PGY 2	5	26.32%
PGY 3	8	42.11%
PGY 4	1	5.26%
Fellow	1	5.26%
Attending	1	5.26%
Total	19	100.00%

## Discussion

CHAOS in the ED was successful. The organizers achieved their goal of enhancing medical education using a novel approach to simulation through the creation of a multi-faceted, skills-based competition that tested players’ knowledge, psychomotor skills, and teamwork. Topics spanned a variety of EM content areas.

The variety of skills and knowledge interwoven into each challenge created a relatively new approach to medical simulation, as traditional simulation scenarios usually focus on a single procedural skill or disease process. Feedback from participants was positive. Both strengths and weaknesses were identified. The design also facilitated the sharing of different methods of teaching, as was best exemplified when one resident shared that he intended to incorporate the radiograph review and splinting training into his residency program’s monthly simulation workshop.

The structuring of simulation teams in a random fashion was trialed in this intervention, which included individuals at different levels of training (i.e., students, residents, and faculty). Randomization added an additional layer of complexity, creating teams with members who have never worked with one another. This design encouraged them to learn how to quickly work together and develop trust. To the best of our knowledge, this randomized and multi-tiered educational approach for team creation has not been described on such a large scale. Team formation represented an authentic mechanism for how teams assemble in the real world setting of the emergency department.

Several challenges were identified, and planning is already underway to host a similar competition at CORD 2019. One of the biggest challenges was ensuring a sufficient number of game participants. In the days leading up to the event, organizers recruited individuals interested in competing while at the conference. Those interested had to fill out a ticket that asked for demographic information, as well as a board-style medical question provided by PEER. Correctly answered tickets were placed in a lottery drum on the day of the event for selection. Initially, organizers planned on selecting tickets from the drum at random to assign the teams; however, on the morning of the event, the number of participants presented did not match the tickets. Teams were, therefore, created in true form, “on the fly”.

The event was held on the last day of the CORD conference, as well as during a major session that required the attendance of many conference attendees. Scheduling is crucial when holding such a large-scale event. In addition, by the end of the conference, many attendees had already departed. Guaranteeing a spot to residents may have allowed them to better plan their stay. Moving forward, the organizers will ensure that the competition is scheduled earlier in the conference, not in conflict with any other major events. Although the randomized, “chaotic” method of team creation is a crucial consideration, it would be more convenient for participants to know in advance if they were going to be competing in the competition.

Another challenge experienced was associated with the complexity of some of the challenges, most notably, Round 1A and 1B. These rounds had complex scoring systems, grading participants on a variety of skills and critical actions. During the event the authors quickly realized that these complex rounds were much more difficult to execute, leaving some participants confused. Rounds 2 and 3 were simpler in structure. When attempting to replicate this competition in the future, challenges should only cover one or two content areas. Rounds should also be shorter in duration, ideally less than 10 minutes.

Another minor problem encountered was problems with slow internet speed, as network connectivity was required for the SonoSim equipment. Software was stalled secondary to slow internet speeds, likely secondary to the overcrowded convention center network. In the future, it is recommended to have a dedicated internet connection or hotspot, to ensure adequate connectivity for the simulation equipment and software.

## Conclusions

CHAOS in the ED is a medical education skills competition which “gamifies” procedures, knowledge, and skills from a variety of content areas in EM. CHAOS in the ED was successfully piloted and was well received by those who attended CORD 2018. The conceptual framework behind CHAOS in the ED incorporated various gamification concepts into the national medical simulation community, which can be easily adapted at future simulation conferences and/or gatherings. Future versions should incorporate learners’ feedback for successful iterations at local, regional, and national settings.
